# Bubble-Induced Endothelial Microparticles Promote Endothelial Dysfunction

**DOI:** 10.1371/journal.pone.0168881

**Published:** 2017-01-23

**Authors:** Xuhua Yu, Jiajun Xu, Guoyang Huang, Kun Zhang, Long Qing, Wenwu Liu, Weigang Xu

**Affiliations:** Department of Diving and Hyperbaric Medicine, Faculty of Naval Medicine, the Second Military Medical University, Shanghai, China; Max Delbruck Centrum fur Molekulare Medizin Berlin Buch, GERMANY

## Abstract

Decompression sickness is a systemic pathophysiological process caused by bubbles and endothelial microparticles (EMPs) are established markers reflecting competency of endothelial function and vascular biology. Here, we investigated the effects of bubble-induced EMPs on endothelial cells in vitro and vivo. Rat pulmonary microvascular endothelial cells (PMVECs) were isolated and stimulated by bubbles and bubble-induced EMPs were collected and incubated with normal PMVECs in vitro. Cell viability and apoptosis were detected using Cell Counting Kit-8 assay and Annexin V FITC/PI double staining, respectively. Cell permeability and pro-inflammatory cytokines were determined by electric cell substrate impedance sensing and enzyme-linked immunosorbent assay, respectively. Intracellular nitric oxide and reactive oxygen species production were analyzed microscopically. In vivo study, bubble-induced EMPs were intravenously injected to the rats and soluble thrombomodulin, intercellular adhesion molecule 1, and vascullar adhesion molecule 1 were involved in evaluating endothelial dysfunction. In our study, bubble stimulus resulted in a significant increase of EMPs release by 3 fold. Bubble-induced EMPs significantly decreased cell viability and increased cell apoptosis. Moreover, bubble-induced EMPs induced abnormal increase of cell permeability and over-expression of pro-inflammatory cytokines. Intracellular ROS production increased while NO production decreased. These negative effects caused by bubble-induced EMPs were remarkably suppressed when EMPs pretreated with surfactant FSN-100. Finally, intravenous injection of bubble-induced EMPs caused elevations of soluble thrombomodulin and pro-inflammatory cytokines in the circulation. Altogether, our results demonstrated that bubble-induced EMPs can mediate endothelial dysfunction in vitro and vivo, which can be attenuated by EMPs abatement strategy. These data expanded our horizon of the detrimental effects of bubble-induced EMPs, which may be of great concern in DCS.

## Introduction

Microparticles (MPs) are submicron vesicles (0.1–1.0 μm in diameter) resulting from apoptotic or activated cells, harboring cell surface proteins, cytoplasmic and nuclear constituents, and expressing specific surface markers of the parent cell, which can be utilized to detect the number and origin of MPs [1]. MPs, once they pinch off from the parent cell, can transfer the contents to the targeted cells and MPs from different origins or stimuli can lead to distinct phenotypic characteristics and functional effects [2]. Endothelial microparticles (EMPs) are released from the injured ECs and numerous studies have linked EMPs with many different vascular diseases, such as severe hypertension [3], acute coronary syndromes [4], acute lung injury [5]. During activation and apoptosis, endothelial cells release phenotypically and quantitatively distinct EMPs. Besides the verification of phosphatidylserine (PS) on EMPs, CD144 appear to be the best combination of antigens that suggests a true EMP population. In addition, CD31 and CD105 were markedly increased on EMPs produced via apoptotic stimuli, while CD54, CD62E and CD106 were increased on EMPs during activation [6]. More importantly, EMPs not only constitute an emerging marker of endothelial dysfunction, but also are considered to play a major biological role in inflammatory response, coagulation, angiogenesis, and thrombosis [7–9].

Decompression sickness (DCS), as a pivotal medical problem in diving, is caused by intravascular bubbles that are formed as a result of reduction in ambient pressure [10]. The central role of bubbles as an inciting factor for DCS is widely accepted and several studies have focused on the role of bubbles and subsequent inflammatory response [11, 12]. Some studies in vitro have demonstrated that cell activity and function impaired after bubble contact with the ECs [13, 14]. Similarly, in the pathophysiological process of DCS, bubbles can contact with the ECs and subsequently cause endothelial dysfunction [15–17]. Moreover, erythrocytes, leukocytes and platelets are activated and the level of MPs elevated in DCS model [18]. Intravenous injection of decompression-induced MPs to mice resulted in neutrophil activation and subsequent vascular injuries, which prompted that the MPs play an important role in progress of DCS. Thus several studies in vitro have confirmed that EMPs derived from different origins could induce endothelial dysfunction [9, 19].

Given that these data suggested that bubble contact could impair the ECs, but there have been no direct evidences showing that the injury was accompanied by EMPs release. Elevation of decompression-induced MPs in circulation can lead to a series of inflammatory responses, but the section of EMPs is still unclear. We hypothesized that EMPs caused by bubble stimulus contributed to endothelial dysfunction and the progress of DCS. Therefore, the present study aims to investigate the potential adverse effects of bubble-induced EMPs on ECs in vitro and in vivo.

## Materials and Methods

### Cell culture of PMVECs

**Fig 1:**
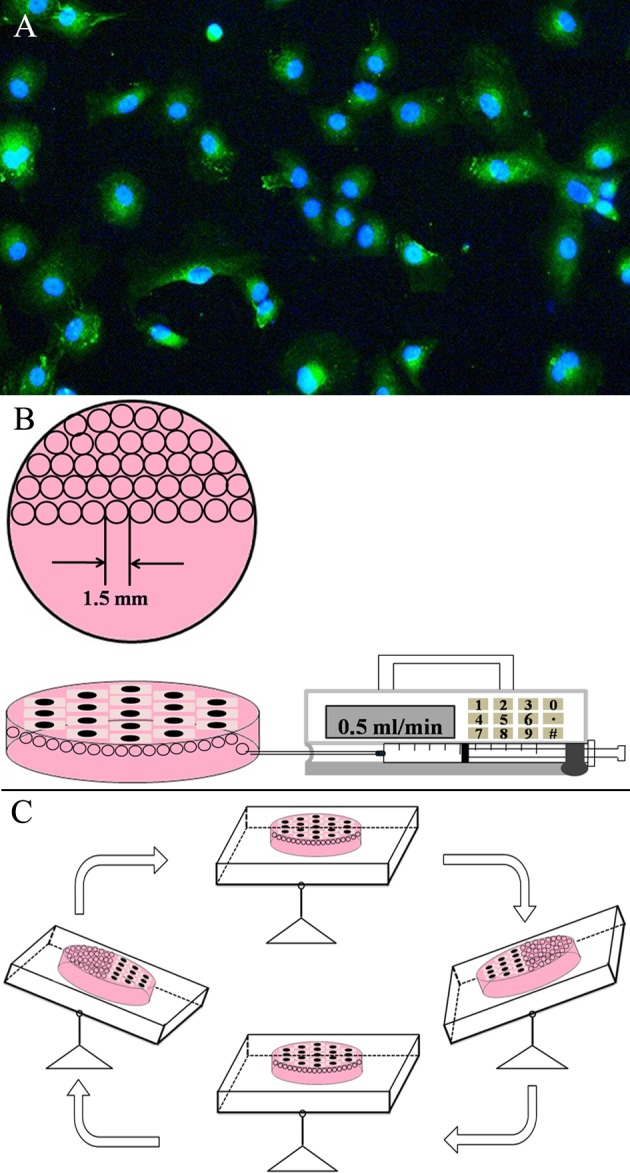
EMPs derived from PMVECs. (A) Primary culture of PMVECs. Representative immunofluorescence images of PMVECs immunestained with antibody against von Willebrand factor. (B) One 35 mm diameter dish filled with CO_2_-independent medium was converted and half of one milliliter nitrogen was injected into the device. (C) The device was put into a shaking table allowing bubbles to contact with the PMVECs.

Five-week-old male Sprague-Dawley rats purchased from SLAC laboratory animal company (Shanghai, China) were housed at constant temperature (23±1°C) and humidity (55±5%) with a 12-h light/dark cycle. Rat pulmonary microvascular endothelial cells (PMVECs) were isolated according to a modified method previously used by Magee et al [20]. The lungs were removed and placed in a conical flask containing M199 with 0.005% antibiotic solution. The visceral pleura were first detached by pre-warmed 0.25% trypsin and the outer 1–2 mm of peripheral lung tissue was dissected and finely minced into 1 mm^3^ slices in a dish at 4°C. The slices were digested with 0.25% collagenase IV at 37°C for 30 min and subsequently with 1% dispase at 37°C for 30 min. Enzymes were inactivated with Dulbecco’s modified Eagle’s medium (DMEM, Invitrogen) supplemented with 20% fetal bovine serum (FBS, Gibco) and supernatant was removed after centrifuged at 600g for 10 min. Cell suspensions were seeded on polylysine-coated 75-cm^2^ culture flasks (Corning, USA) and cultured at 37°C in a humidified atmosphere of 5% CO_2_. Complete Rat Endothelial Cell Medium (CRECM, Cell Biologics) was changed every 3 days and subculture was performed when PMVECs reached confluence. Identification of cells possessing factor Ⅷ antigen was done using immune fluorescence with goat anti-human von Willebrand factor antiserum as the primary antibody (Fig 1A).

### EMPs generation

PMVECs were digested and cultured on a 35 mm diameter dish (Corning, USA), with 5×10^5^ in it. Cells were allowed to attach and grow to monolayer in the incubator for 12 h. The CRECM was exchanged to CO_2_-independent medium (Gibco, USA) before bubble stimulus to sustain the pH. Half of one milliliter nitrogen (N_2_) was injected to the medium and the device was put into a 37°C shaking table so that bubbles were allowed to contact with the confluent cells for 30 min (Fig 1B). The culture medium was collected and cleared of cells and cell debris by centrifugation at 10000g at 4°C for 30min. This protocol was established on the basis of pilot trials focused on eliminating cell debris, which was expected to be elevated by bubble stimulus. Subsequently, the supernatant was subject to ultracentrifugation at 100000g at 4°C for 60min to obtain EMPs pellet. EMPs pellet was resuspended in 200 μl of 0.1-μm filtered medium for further study. EMPs from culture medium in normal condition were isolated using the same method and these EMPs were further applied as the normal control group.

### Flow cytometry analysis of EMPs

**Fig 2:**
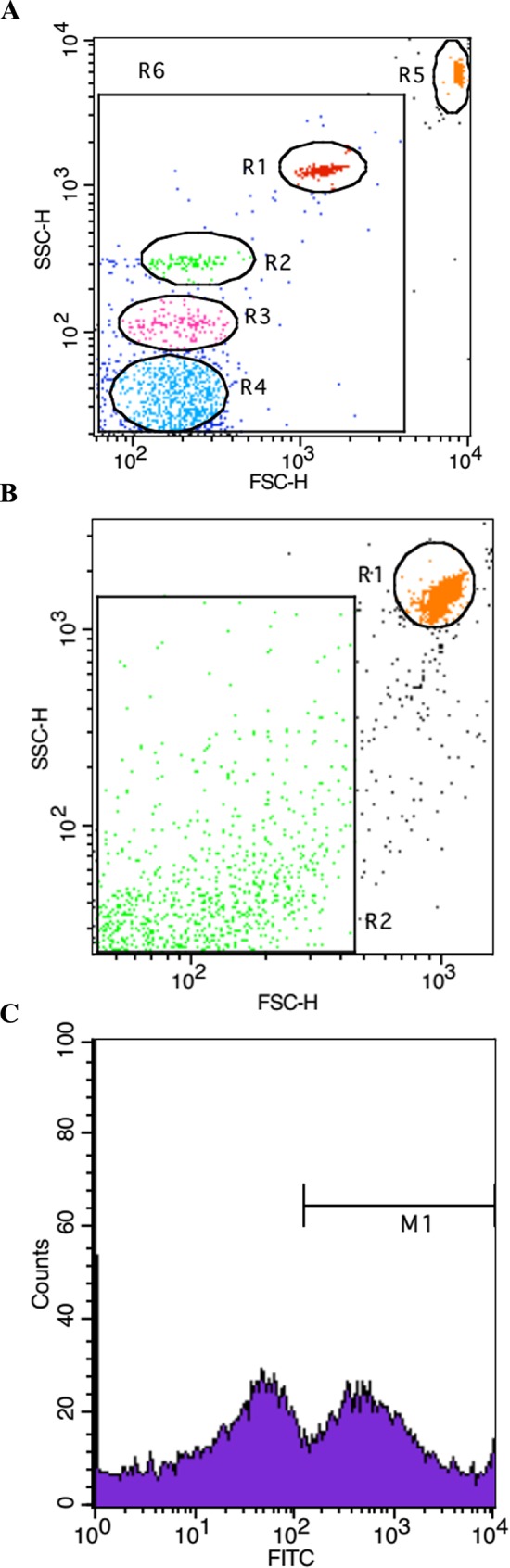
Flow cytometry was used to quantify EMPs. (A) Nano Fluorescent Size Standard Kits (NFPPS-52-4K) to calibrate the MPs. (B and C) Representative flow cytometer image of bubble-induced EMPs (Annexin V +).

In order to eliminate the contamination with exosomes (<100 nm), Nano Fluorescent Size Standard Kits (NFPPS-52-4K) (Spherotech, USA) were applied to calibrate the MPs (0.1–1μm). As showed in Fig 2A, the beads with different sizes (0.22, 0.45, 0.88, 1.34 μm) were detected and the concentration of MPs can be calculated by ACBP-20-10 particles, which have a given concentration (10^6^/ml). EMPs were incubated with Annexin V-FITC for analysis while EMPs from the rats were double-stained with Annexin V-FITC and anti-CD144-PE (Santa Cruz) [6, 21]. The antibody was centrifuged prior to staining. For single analysis, 5 μl of monoclonal antibody or isotype was added to 50 μl of re-suspended pellet tubes and then mixed in 400 μl phosphate buffer solution (PBS). The tubes were incubated for additional 20 min at room temperature and 50 μl of ACBP-20-10 particles (Spherotech, USA) were added to the suspension. Analysis of EMPs was performed using a FACScan flow cytometer (Becton–Dickinson Biosciences, San Jose, CA). Data from 2000 events were acquired (Fig 2B and 2C) and the absolute number of EMPs was calculated using the following formula:

$$Absolute\;number\;of\;EMPs/\mu l=\frac{\#\;of\;events\;in\;EMPs\;regien}{\#\;of\;beads\;collected}\times \frac{\#\;of\;beads\;per\;tube}{volume\;of\;sample\;initially\;used}$$

### EMPs abatement strategy

The EMPs were made 33% (200 μl of particles suspension combined with 100 μl of FSN-100, DuPont, USA) and incubated at 37°C for different point-in-time (0.5 h, 1 h, 2 h).The number of EMPs was analyzed according to protocol aforesaid.

### Co-incubation of the EMPs with PMVECs

Four groups were set: the normal control group, the FSN-100 group, the EMPs-treated group and the EMPs+FSN-100 group. EMPs pellet was resuspended in DMEM (EMPs suspension) and added to the PMVEC cultures at a final concentration of 5×10^5^/ml (EMPs group). EMPs suspensions at the same concentration were made 33% with 0.3% FSN-100 and then added to PMVEC culture (EMPs-FSN-100 group). In FSN-100 group, 0.3% FSN-100 was added to the culture with the same proportion. All of the groups were incubated for 12 h and the indices undermentioned were involved in evaluating the effects of EMPs.

### CCK-8 and Annexin V FITC/PI

PMVECs were seeded at the density of 1×10^5^ cells/ml into 96-well plates with the volume100 μl or into 6-well plates with the volume 2ml. Cell viability was detected using Cell Counting Kit-8 (CCK-8, Dojindo, Japan) solution following the instructions and then the absorbance at 450 nm was measured. PMVECs in 6-well plates were used for apoptosis test. In brief, collected cells were washed twice with PBS and resuspended in 1×Binding Buffer and stained with Annexin V FITC and PI dyes. Cells were then analyzed with flow cytometry (Beckman Coulter, USA).

### Transendothelial electrical resistance

Transendothelial electrical resistance (TEER) of PMVEC monolayer was determined using Millicell ERS-2 (Millipore Corporation, Billerica MA) according to the instruction manual. PMVECs were seeded at the density of 1×10^5^ cells/cm^2^ on collagen-coated (10 μg/cm^2^) transwell polycarbonate filters (pore size = 0.4 μm, exposed area = 1.1 cm^2^, France). The resistance was recorded and each experiment had three replicates and was repeated three times. Unit Area Resistance was calculated using the following formula:

$$Unit\;Area\;Resistance=Resistance\;\left(\Omega \right)\times Effective\;Membrane\;Area\;\left(cm^{2}\right)$$

### Enzyme-linked immunosorbent assay (ELISA)

The cultural solutions were collected and centrifuged at 1500 g at 4°C for 15 min. The supernatant was collected and stored at -80°C for further study. Soluble ICAM-1 and soluble VCAM-1 (Jiancheng Bioengineering Institute, Nanjing, China) were determined according to their instructions.

### In Situ Measurement of NO and ROS

NO and ROS content was imaged using DAF-FMDA or DCFH-DA under epifluorescence microscopy. Roots were incubated with 5 μM DAF-FMDA or 10 μM DCFH-DA for 20 min at 37°C, washed three times with PBS to wipe out the remnant DAF-FM DA or DCFH-DA, and analyzed microscopically (Nikon Eclipse 80i, Nikon, EX 460–500, DM 505, BA 510–560).

### Soluble thrombomodulin (s-TM) and pro-inflammatory cytokines detection

Sprague-Dawley rats (250–280 g) were randomly divided into four groups: NC, FSN-100, EMPs and EMPs+FSN-100 group and then intravenously injected into the tail vein. The experiment protocol was approved by the Animal Ethics Committee of the Second Military Medical University and the procedures were carried out in compliance with related guidelines and regulations. After 24 h, all of the rats were anesthetized with 3% pentobarbital sodium (1.5 ml/kg body weight, i.p.), and no rats suffered severe pain. Peripheral blood was obtained and serum levels of s-TM, s-ICAM-1 and s-VCAM-1 (Jiancheng Bioengineering Institute, Nanjing, China) were assayed using double antibody sandwich assay of ELISA according to their instructions.

### Statistical analysis

Data are shown as mean±SD. The student’s t-test or Mann Whitney test was used to determine statistical significance between two comparable parameters. In the case of multiple parameters, the ANOVA test was applied. Values of P < 0.05 were considered significant.

## Results

### Bubble stimulus caused an elevation of EMPs release

**Fig 3:**
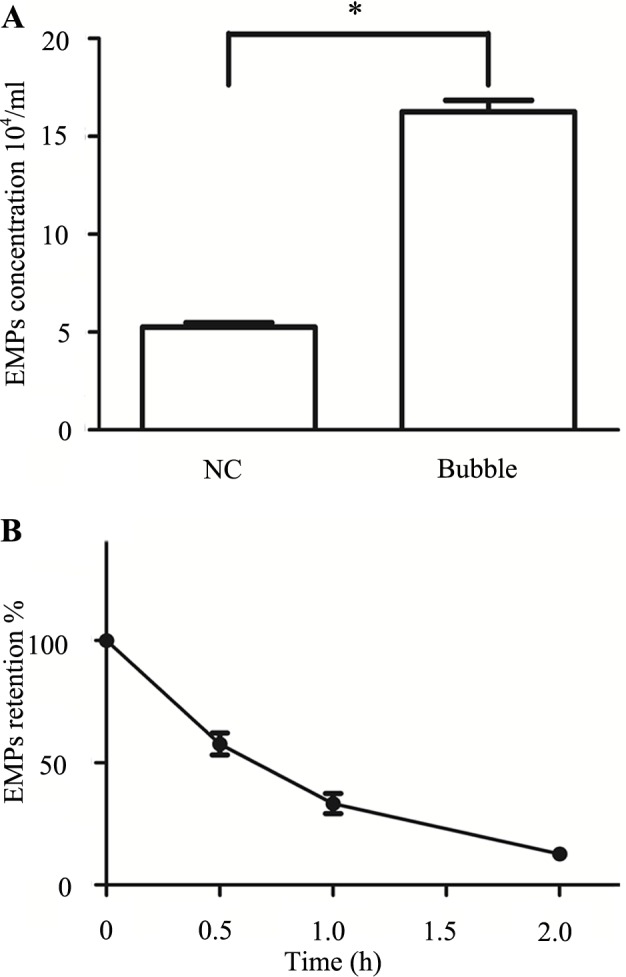
Bubble-induced EMPs release and EMPs abatement strategy. Values are means ± SD (n = 3). (3A) EMPs from cultural medium 1 h after bubble stimulus. (3B) EMPs combined with FSN-100 and incubated for 0–2 h at room temperature. *P < 0.05.

Confluent PMVECs were contacted with the N_2_ bubbles for 30 min, and the cultural medium was obtained at 1 h post. MPs were identified on the basis of size and Annexin V. There was a significant difference in groups with EMPs in bubble treated group being markedly elevated about 3-fold compared with the normal control group (Fig 3A).

### Abatement strategy of EMPs

The level of EMPs had a notly decline when EMPs incubated with 0.3% (w/vol) FSN-100 for 2 h (S1 File) and there was no significant difference with the normal control group (Fig 3B).

### Bubble-induced EMPs impaired endothelial activity

**Fig 4:**
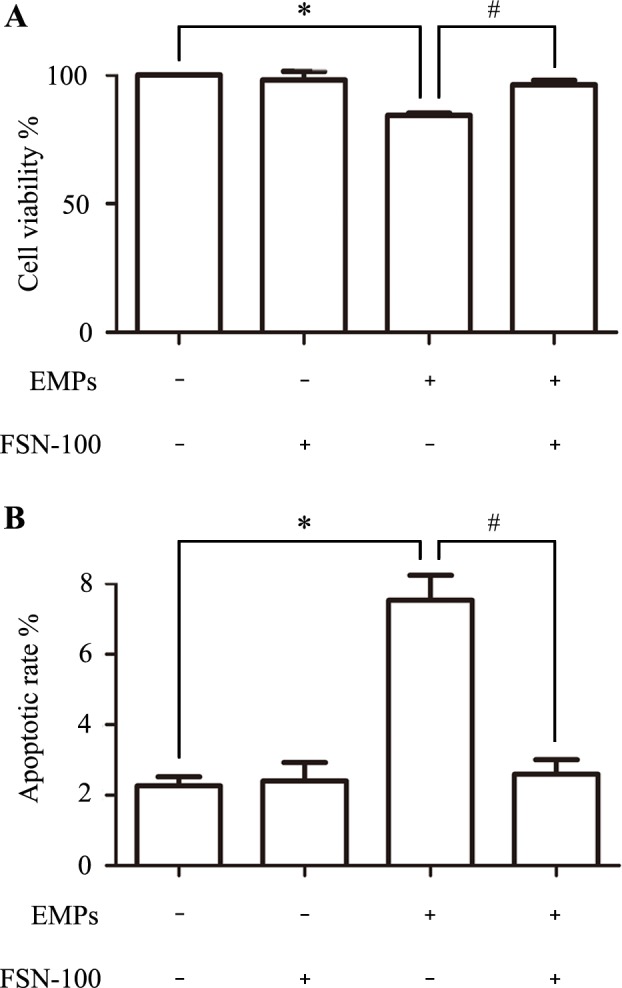
Changes of cell activity after incubating with EMPs for 12 h. Values are means ± SD (n = 3). (A) Cell viability decreased 14% after EMPs treatment and FSN-100 abatement can attenuate this effect. (B) Cell apoptosis increased after EMPs treatment and FSN-100 abatement can attenuate this effect. *P < 0.05, # P < 0.05.

In EMPs-treated group, cell viability decreased about 14% compared with the normal control group (S2 File) and this effect disappeared when EMPs pretreated with FSN-100 for 2 h (Fig 4A). Apart from this, cell apoptotic level significantly elevated (S3 File) in EMPs-treated group but not in EMPs-FSN-100 treated group (Fig 4B). Moreover, cell viability and apoptosis unchanged in FSN-100-treated group, indicating that the surfactant FSN-100 couldn’t do harm to endothelial activity.

### Bubble-induced EMPs promoted pro-inflammatory cytokines release

**Fig 5:**
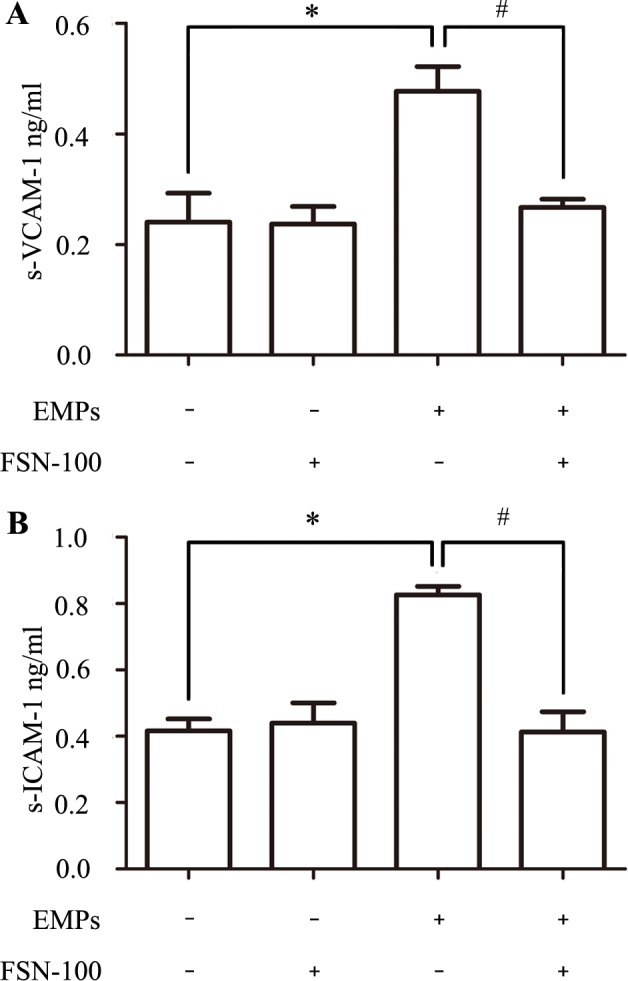
Normal PMVECs were incubated with bubble-induced EMPs and proinflammatory cytokines were analyzed. Values are means ± SD (n = 3). (A) The concentration of soluble VCAM-1 increased about 2 times compared with the normal control group (0.48 ± 0.05 ng/ml vs. 0.24 ± 0.05 ng/ml). (B) Soluble ICAM-1 in EMPs-treated group was 0.83 ± 0.03 ng/ml while 0.42 ± 0.04 ng/ml normal control group. *P < 0.05, # P < 0.05.

Bubble-induced EMPs promoted the release of s-VCAM-1 (S4 File) with the concentration of EMPs-treated group being about 0.48±0.05 ng/ml and normal control treated group being 0.24±0.05 ng/ml (Fig 5A). Besides, the concentration of s-ICAM-1 increased about 2 times (S5 File) compared with the normal control group (Fig 5B). Finally, the level of s-ICAM-1 and s-VCAM-1 didn’t enhance in EMPs+FSN-100 group compared with the EMPs group, nor in FSN-100 group compared with the normal control group.

### Bubble-induced EMPs decreased TEER

**Fig 6:**
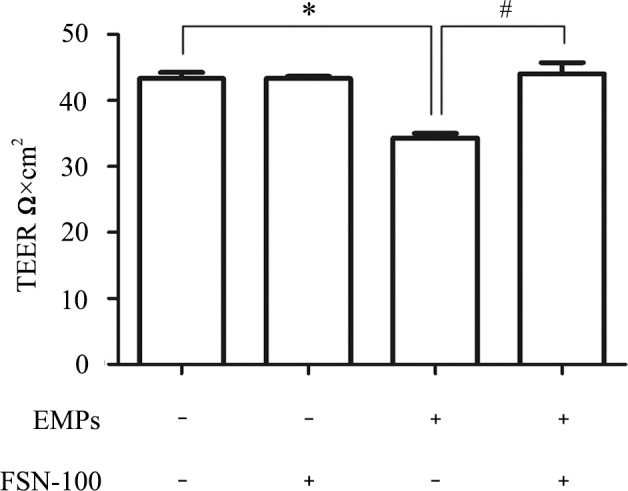
TEER changes after bubble-induced EMPs treatment. Monolayer permeability of PMVECs increased in EMPs treated group (34.3 ± 1.2 Ω vs. 43.3 ± 1.5 Ω) but not in EMPs+FSN-100 treated group (44 ± 3 Ω vs. 43.3 ± 1.5 Ω). *P < 0.05, # P < 0.05.

The results showed that Unit Area Resistance of EMPs group (34.3±1.2 Ω) was significantly lower than in the normal control group (43.3±1.5Ω) (Fig 6), suggesting that the permeability of PMVECs significantly increased after bubble-induced EMPs treatment. Besides, TEER value was significantly higher in EMPs+FSN-100 group (44±3 Ω) than in EMPs group (S6 File).

### EMPs impaired NO production accompanied with ROS augment

**Fig 7:**
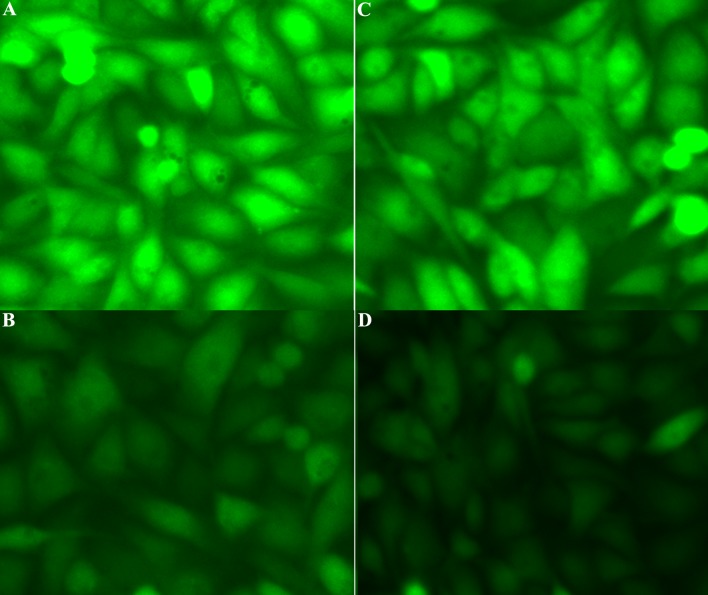
Intracellular NO and ROs production after bubble-induced EMPs treatment. Image of NO production in EMPs treated PMVECs (B) and normal control group (A). Image of ROS production in EMPs treated PMVECs (C) and normal control group (D).

To determine whether the production of NO and ROS play a role in the biological actions of bubble-induced EMPs, intracellular NO and ROS production were detected using DAF-FMDA or DCFH-DA under epifluorescence microscopy. As showed in Fig 7, NO intensity in EMPs-treated group was significantly lower than in normal control group while ROS intensity significantly higher.

### Endothelial changes in vivo study

**Fig 8:**
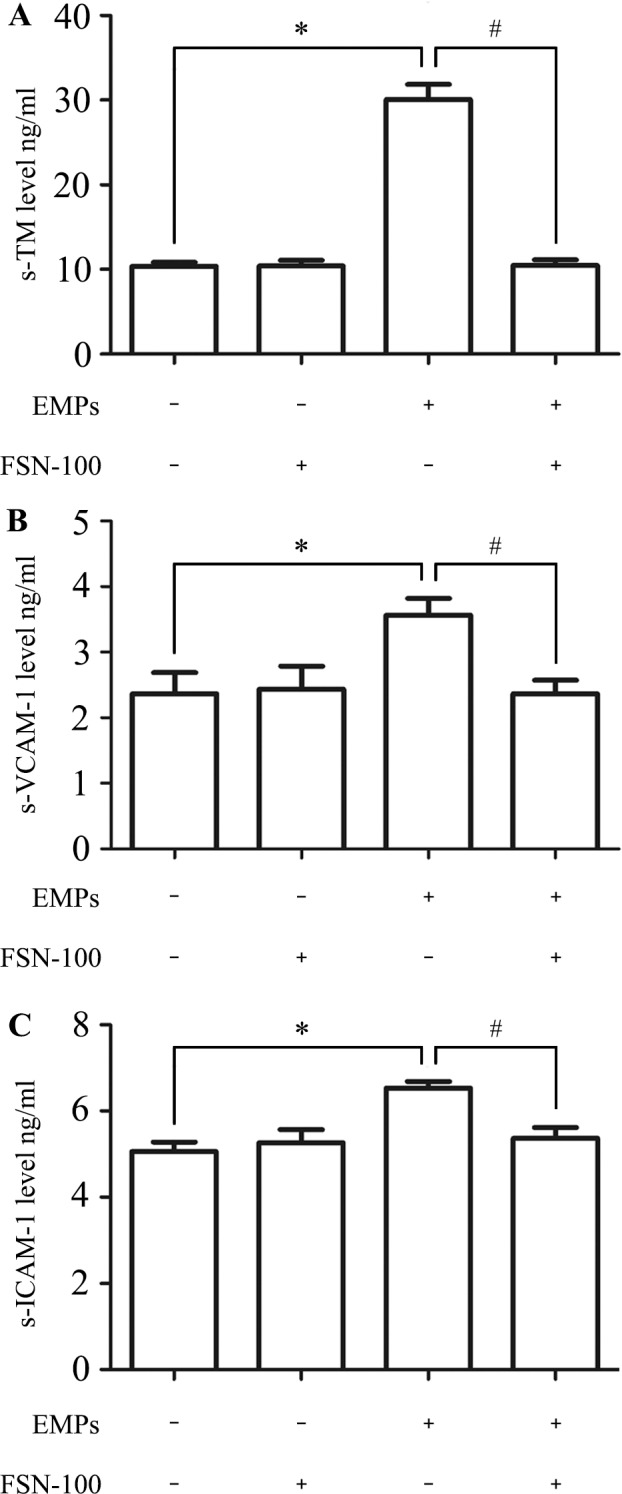
Serum levels of s-TM s-ICAM-1 and s-VCAM-1 were detected to evaluate endothelial injury in vivo. Values are means ± SD (n = 3). (A) The concentration of s-TM increased about 3 times in EMPs treated group compared with the normal control group. (B) The level of s-VCAM-1 was ng/ml in EMPs treated group and ng/ml in normal control group. (C) The level of s-ICAM-1 was times higher in EMPs treated group than in normal control group. *P < 0.05, # P < 0.05.

To evaluate whether bubble-induced EMPs can lead endothelial injury in vivo, bubble-induced EMPs were administrated intravenously to SD rats. Soluble TM level in serum increased about three times (S7 File) than in the normal control group (30.23 ± 2.55 vs. 10.43 ± 0.57 ng/ml, Fig 8A). Besides, the levels of s-VCAM-1 and s-ICAM-1 significantly increased in EMPs group (S8 and S9 Files) compared with the normal control group (3.57 ± 0.25 vs. 2.34 ± 0.32 ng/ml, 6.53 ± 0.15 vs. 5.07± 0.21 ng/ml, Figs 8B and 7C, respectively), which was consistent with the results in vitro study.

## Discussion

The present study demonstrates that bubble-induced EMPs cause severe endothelial injury, including cell activity and function. This suppressed effect is specific to bubble-induced EMPs, because those EMPs obtained from normal control group can’t mediate endothelial dysfunction.

Endothelium is regarded as the largest organ in the body and plays an important role in many regulatory responses. The reason that ECs receive so much attention in DCS is that they act as a “target organ” of bubbles [12, 22]. Normally, little or no decompression-induced bubbles should pass through the pulmonary circulation after DCS occurrence, but the MPs could. A single air dive can lead to arterial endothelial dysfunction even with few detected vascular bubbles [23] and progressive elevations in circulating MPs have been demonstrated after decompression stress, which can further induce neutrophil activation and subsequent vascular injuries [17]. Such evidences indicated that MPs may act as a hazard and contribute to the deterioration of DCS.

EMP is regarded as an indicator of endothelial injury and our results are consistent to previous studies demonstrating that bubble contact with endothelium can result in cell death in response to calcium influx and mitochondrial depolarization [12]. EMPs represent a relatively small (5–15%) but very important subset of all circulating microparticles and this proportion vary indifferent inflammatory or vascular diseases, reflecting the interaction of pathological process with endothelium[24, 25].

Several studies in vitro have confirmed that EMPs could mediate endothelial dysfunction, such as decline of angiopoiesis, vasorelaxation, nitric oxide production, proliferation and migration [8, 18, 26]. In our study, a pure population of bubble-induced EMPs was used and endothelial activity and function were injured after co-incubation with them. Cell viability and cell apoptosis are frequently used as direct indicators reflecting cell activity. Clinical manifestations also indicated increased vascular permeability related to endothelial dysfunction in DCS [27] and in this study we demonstrated that bubble-induced EMPs significantly decreased TEER value in PMVECs, indicating a higher permeability.

Both reactive oxygen species (ROS) and Nitric oxide (NO) contribute to the homeostasis of vascular functions, including vascular tone, endothelial cell proliferation and apoptosis [28]. In our study, intracellular ROS and NO levels are involved to evaluate whether bubble-induced EMPs could inhibit endothelial function. When ROS generation exceeds the capacity of antioxidant defense, progressive endothelial dysfunction and occur [29]. NO is an important signaling molecule that regulates vascular relaxation, angiogenesis, and thrombosis. NO bioavailability is critical for the maintenance of vascular physiology and it is a potent vasodilator produced by endothelial nitric oxide synthase activity in endothelial cells [30].

Both ICAM-1 and VCAM-1 express on the ECs and they play an important role in leukocyte adhesion and subsequent inflammation. Besides, it has been confirmed that decompression-induced MPs also express the specific makers of ICAM-1and VCAM-1. Moreover, some studies have demonstrated that EMPs have the ability to induce the release of many proinflammatory cytokines, including ICAM-1 and VCAM-1. Thus, we anticipated the level of s-ICAM-1 and s-VCAM-1 would be upregulated following EMPs treatment and may contribute to endothelial dysfunction. Moreover, we further observed the effects of bubble-induced EMPs on endothelium in vivo. The presence of s-TM has been recognized as a direct marker of vascular damage. Usually absent in the blood of healthy individuals, the level of s-TM elevated in many inflammatory and thrombotic disorders [31].

Eckmam et al have demonstrated that surfactant Pluronic F-127 can mitigate endothelial cell death caused by bubble contact [32]. Thom et al also found that MP abatement strategy PEG-TB can reduce decompression-induced intravascular neutrophil activation, neutrophil sequestration, and tissue injury. FSN-100 is a water-soluble, ethoxylated nonionic fluorosurfactant that contains no solvent, which confirmed to be harmless to cell activity and function in our study. EMPs pretreated with FSN-100 couldn’t induce these adverse effects aforesaid, which further indicated that bubble-induced EMPs per se lead the injury.

Indeed, questions persist regarding the mechanisms for bubble-induced EMPs production and the present model can’t completely stimulate what happens to endothelium in DCS. Bubble-induced EMPs may only occupy a section of all EMPs after DCS occurrence. Other factors such as inflammatory response may also activate the endothelium and subsequently induce the EMPs release. Further work will be needed to determine the pathway in which bubbles induce EMPs release. Moreover, the relevant inhibitors will be applied in vitro to verify whether the potential methods were effective or not.

All in all, our present results provided evidences that bubble-induced EMPs can lead to endothelial injury, which demonstrate that bubble-induced EMP may be a potential morbigenous factor in the development of DCS and suggest a possible vicious cycle: elevated number of EMPs is a marker of endothelial injury which in turn aggravates endothelial dysfunction in DCS.

## Supporting Information

S1 File Bubble-induced EMPs and EMPs retention after FSN-100 treatment. (XLSX)

S2 File Cell viability after EMPs treatment. (XLSX)

S3 File Apoptotic rate after EMPs treatment. (XLSX)

S4 File s-VCAM-1 concentration after EMPs treatment. (XLSX)

S5 File s-ICAM-1 concentration after EMPs treatment. (XLSX)

S6 File TEER after EMPs treatment. (XLSX)

S7 File serum level of s-TM after injection of EMPs. (XLSX)

S8 File Ferum level of s-VCAM-1 after injection of EMPs. (XLSX)

S9 File Ferum level of s-ICAM-1 after injection of EMPs. (XLSX)
